# Incorporating Nanopore Sequencing Into a Diverse Diagnostic Toolkit for Incontinentia Pigmenti

**DOI:** 10.1155/humu/6657400

**Published:** 2025-01-30

**Authors:** Simone Ahting, Denny Popp, Henry Oppermann, Vincent Strehlow, Maria Fasshauer, Bernt Popp, Maike Karnstedt, Isabell Schumann

**Affiliations:** ^1^Institute of Human Genetics, University of Leipzig Medical Center, Leipzig, Germany; ^2^Center for Pediatric and Adolescent Medicine, St. Georg Hospital Leipzig, Leipzig, Germany; ^3^IDCL (ImmunDefectCentrum Leipzig), St. Georg Hospital Leipzig, Leipzig, Germany; ^4^Center of Functional Genomics, Berlin Institute of Health at Charité, Berlin, Germany; ^5^Department of Medical Genetics, Centre of Medical Genetics, University of Münster, Münster, Germany

## Abstract

Incontinentia pigmenti (IP) is a rare hereditary disorder affecting 1.2 in 100,000 live births, predominantly females. Genetic analysis of IP is complicated by a homologous pseudogene, making conventional short-read sequencing challenging. While long-range PCR is typically used to overcome this, skewed X-inactivation detection can also aid in assigning variants to *IKBKG*. We employed a comprehensive approach, incorporating whole-exome sequencing (WES), long-range PCR, RT-PCR, X-inactivation analysis, and nanopore sequencing, to identify and accurately phase a small heterozygous deletion, NM_001099857.5: c.363_367del, p.(Leu122Glyfs⁣^∗^14), in the *IKBKG* gene in an IP-affected family. The deletion was initially detected via WES, with skewed X-inactivation observed in both the proband and her mother. Long-range PCR specific to *IKBKG* confirmed the variant's location in the *IKBKG* gene, not in the pseudogene. On the RNA level, the variant was undetectable, suggesting nonsense-mediated decay of the transcript. Nanopore sequencing precisely mapped the variant to *IKBKG* and analyzed the methylation status of both alleles, confirming the skewed X-inactivation, with the variant-carrying allele predominantly inactivated. This demonstrates the nanopore sequencing's value in genetic diagnosis, enabling precise variant localization and analysis of X chromosome activation status in females with skewed X-inactivation, aiding in accurate diagnosis and understanding of IP.

## 1. Introduction

Incontinentia pigmenti (IP) (Bloch-Sulzberger-syndrome, OMIM #308300) is a rare hereditary multisystemic neuroectodermal disorder caused by pathogenic variants in the *IKBKG* gene (formerly known as *NEMO* (nuclear factor-kappa B (NF-kB) essential modulator)) on X chromosome. It occurs in 1.2 out of 100,000 live births [1], mainly affects females, and is generally lethal in males during embryogenesis [2], with the rare exception of cases with somatic mosaicism or XXY karyotype [3]. IP is characterized by typical skin manifestations along the Blaschko lines shortly after birth that can be divided into four successive inflammatory stages from blisters and pustules on the extremities (Stage 1), to verrucous lesions (Stage 2), hyperpigmentation (Stage 3), and hypopigmentation (Stage 4) that often persists into adulthood [4]. Ophthalmologic, odontologic, and neurologic impairment can occur additionally as systemic manifestation of IP. In females with heterozygous pathogenic *IKBKG* variants, cells expressing the mutated allele are usually selected against early in life, leading to extremely skewed X chromosome inactivation (XCI) [5] that can serve as a diagnostic criterion for IP. This elimination of mutant allele-expressing cells is due to the vital role of *IKBKG* in the activation of NF-kB pathways that are crucial for many physiological functions, such as immune, inflammatory, antiapoptotic, and developmental pathways [6]. Cells lacking the *IKBKG*-encoded NEMO/IKK*γ* protein due to loss-of-function variants show increased sensitivity to proapoptotic stimuli [7] hence driving skewing of XCI. Traditionally, this can be investigated through analysis of methylation status on CpG sites of two polymorphic X-chromosomal repetitive elements near the *AR* and the *RP2* locus, which can be used for separation of the parental alleles [8, 9].

**Figure 1:**
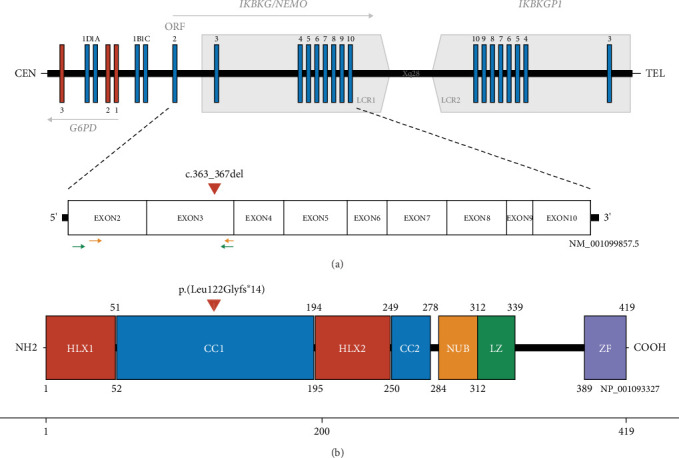
The *IKBKG* locus on chromosome X (adapted from Pescatore et al. [12]). (a) Schematic representation of the Xq28 locus including *IKBKG*, its pseudogene *IKBKGP1*, and the NM_001099857.5 transcript. The square grey arrow boxes represent the low copy repeats (LCR1/2) of the segmental duplication, the exons of *IKBKG/IKBKGP1* are depicted in blue, and the exons of *G6PD* in red. The *IKBKG* gene incorporates nine coding exons and four alternative noncoding exons (1A–1D) that overlap with the locus of *G6PD*. Translation initiation site is located at the beginning of Exon 2, marked here with ORF. The transcript is composed of the Exons 2–10; the identified variant c.363_367del in Exon 3 of *IKBKG* is marked with a red triangle. RNA primer pairs used for confirmation of variant location are shown as green and orange arrows (primer pair B = orange arrows, primer pair C = green arrows; see Table S2). (b) Schematic representation of the IKBKG protein, its domains as well as indication of the identified variant p.(Leu122Glyfs⁣^∗^14) in NP_001093327 (419 aa). Domains are as follows: HLX1: helical domain; CC1: coiled coil; HLX2: helical domain; CC2: coiled coil; NUB: NEMO ubiquitin binding; LZ: leucin zipper; ZF: zinc finger; aa ranges are indicated below and above the domain.

The *IKBKG* gene (NM_001099857.5) on the Xq28 locus is 23 kilobases (kb) long and consists of nine coding exons (Exons 2–10), an additional four alternative noncoding first exons (1A–D), as well as two promoters. The unidirectional Promoter A directs transcription of Exons 1A and 1D and is located in Intron 2 of the *G6PD* gene that is situated on the opposite strand. Promoter B is a strong bidirectional promoter driving transcription of Exon 1B and 1C as well as *G6PD* itself [10]. A nonfunctional second copy of *IKBKG* with > 99% sequence identity [11] comprising the Exons 3–10 makes up the nonprocessed, nontranscribed *IKBKG* pseudogene *IKBKGP1*. Together, they are part of a 35.7-kb segmental duplication that consists of two low copy repeats (LCR1/2) adjacent and in opposite orientation, one covering *IKBKG* and the other one *IKBKGP1* [10] (Figure 1). The most common pathogenic variant accounting for 70%–80% of IP cases is a large deletion spanning the Exons 4–10, termed *IKBKG*del [13]. Other variants pathogenic for IP have been reported and include small delins (54%) and single nucleotide substitutions (46%). The latter are mostly variants that lead to premature termination codons (PTCs), either through nonsense or frameshift changes, while only 15% are missense variants [13]. In fact, owed to the high homology of the *IKBKG* and the *IKBKGP1* loci, intra-locus genomic rearrangements are frequent and have been shown to render the IP locus susceptible to novel pathological alterations [10]. Due to the high similarity of the *IKBKG* and the *IKBKGP1* locus, detection of *IKBKG* variants using short-read sequencing is hindered, since the presence of *IKBKGP1* affects read depth, mapping quality, and alignment, increasing the risk for false positive or false negative results [14]. Therefore, so far, Sanger sequencing using long-range PCRs has been used as the method of choice in IP diagnostics [15].

In our present study, we describe a female individual with characteristic phenotype of IP and a similarly affected mother. We applied whole-exome sequencing (WES), Sanger sequencing, X-inactivation analysis, RT-PCR, and long-read amplicon sequencing to confirm a novel frameshift variant in *IKBKG* in the affected family. Furthermore, we utilized Oxford Nanopore Technology (ONT) sequencing not only to differentiate whether the variant is located in the gene or pseudogene but it also succeeded in ascertaining whether the variant was located on the predominantly active or inactive X chromosome by analyzing the methylation frequency at the two *IKBKG* promoters.

## 2. Material and Methods

### 2.1. Ethics and Consent

This study adheres to the principles set out in the Declaration of Helsinki. The study was approved by the Ethical Committee of the Medical Faculty of the Leipzig University, and the authors received and archived the written consent of the affected individuals and/or their legal guardians for both study participation and publication of genetic and clinical data as well as photographs.

### 2.2. Exome Sequencing

Genomic DNA was isolated from EDTA blood samples of the index and parents using the MagCore HF16 Plus Nucleic Acid Extractor; DNA concentration was measured using a NanoDrop 2000 (Thermo Scientific). Enrichment and library preparation for exome sequencing was performed using the TWIST Human Core Exome Kit (TWIST Bioscience, San Francisco, CA, United States). Libraries were sequenced with 150 basepairs (bp) paired-end reads on a NovaSeq 6000 system (Illumina, Inc., San Diego, CA, United States). On average, coverage of targeted genomic regions was > 100× with 97% covered at least 10×. Raw data was processed using varfeed followed by a tertiary analysis with the browser-based genomics software Varvis (Limbus Medical Technologies GmbH, Rostock, Germany). The variant in *IKBKG* (NM_001099857.5, *MANE*-select) was classified according to the ACMG (American College of Medical Genetics and Genomics) criteria and latest recommendations [16], the ACGS (Association for Clinical Genomic Science) Best Practice Guidelines 2019 [17], and the ClinGen Sequence Variant Interpretation Recommendations for PM2—Version 1.0 [18].

### 2.3. Sanger Sequencing and Multiplex Ligation-Dependent Probe Amplification (MLPA)

Bidirectional Sanger sequencing for confirmation and segregation analysis of the parents was performed on the Applied Biosystems 3500 Genetic Analyzer (Thermo Fisher Scientific Inc., Waltham, Massachusetts, United States), using two sets of primers binding to regions in Exon 3 or adjacent introns (Table S1). The identified variant was submitted to ClinVar [19]. MLPA analysis for the *IKBKG* gene was performed using the P073-B2 kit (MRC-Holland) on an Applied Biosystems 3500 Genetic Analyzer (Thermo Fisher Scientific Inc., Waltham, Massachusetts, United States). Sanger sequences and MLPA were analyzed using Sequence Pilot software (JSI Medical Systems, Ettenheim, Germany).

### 2.4. RNA Analyses

For identification of the variant in *IKBKG* on RNA level, RNA was isolated from peripheral blood lymphocytes using the PAXgene Blood System (Becton Dickinson, Franklin Lakes, NJ, United States) of the individual and the mother. Complementary DNA (cDNA) was generated using PrimeScript RT Master Mix (TaKaRa Bio Europe SAS, Saint-Germain-en-Laye, France) according to the manufacturer's instructions. RT-PCR was conducted followed by bidirectional Sanger sequencing of parts of the Exons 2–4 (Table S2) on the Applied Biosystems 3500 Genetic Analyzer (Thermo Fisher Scientific Inc., Waltham, Massachusetts, United States), and PCR product was visualized by gel electrophoresis. Bioinformatic analysis was done using the Sequence Pilot Software (JSI Medical Systems, Ettenheim, Germany).

### 2.5. Validation of Variant by Long-Range PCR

For validation of the variant as well as differentiation between IKBKG and IKBKGP1, long-range PCR of the IKBKG locus in the index patient, amplification of the respective exon as well as sequencing was performed in an external laboratory.

### 2.6. X-Inactivation

X-inactivation testing on EDTA whole blood was recommended after variant identification for both the index and the mother. To this end, XCI status was evaluated through digest by methylation-sensitive restriction enzyme *Hpa*II followed by PCR amplification of digested and undigested genomic DNA using primers for the highly polymorphic CAG-repeat locus in the *AR* gene [9] as well as the extragenic tandem-repeat-GAAA-locus 5⁣′ of the RP2 promoter [8]. Products were separated using an Automatic Genetic Analyser, and the methylation status was calculated as previously described [20]. Skewing is present when the predominant allele constitutes more than 74% of all alleles, a percentage between 90% and 100% is considered extreme skewing.

### 2.7. Nanopore Sequencing

DNA (3 *μ*g) from EDTA whole blood was prepared for ONT sequencing using the Ligation Sequencing Kit (SQK-LSK114, Oxford Nanopore Technologies) according to the manufacturer's instructions with the following adjustments: Incubation time of the end-prep reaction was increased to 15 min at 20°C and 15 min at 65°C. Incubation time of adapter ligation reaction was adjusted to 30 min at room temperature. The library (300 ng) was loaded onto a PromethION flow cell type R10.4.1 and sequenced on a P2Solo device (ONT). The sequencing run was performed using adaptive sampling targeting the q-arm of the X chromosome as well as the *RP2* locus on the p-arm with 50 kb flanking region up- and downstream. The flow cell was washed twice, once after 18 h and again after a further 29 h of sequencing using Wash Kit EXP-WSH004 (ONT) according to the manufacturer's instructions. After each washing step, another 300 ng of DNA was loaded onto the flow cell.

Raw sequencing data (fast5 files) was basecalled and mapped against the reference human genome hg38 by guppy v6.5.7 (ONT) using a methylation-aware basecalling model with enabled read splitting, adapter trimming, and calibration strand removal. In total, 38.7 Gb of sequencing data was obtained with an on-target read length N50 value of 19 kb (see Table S3 for more details). After exclusion of alignments with a mapping quality below 20, variants were called using Clair3 v1.0.4 [21] (single nucleotide variants (SNVs)), QDNASeq 1.34.0 [22] (copy number variations (CNVs)), and Sniffles2 2.0.7 [23] (structural variants (SVs)). SNVs were phased using WhatsHap v1.7. WhatsHap was further used to assign tags to the reads based on haplotypes and to partition the alignment file into separate haplotypes. For each haplotype, the methylated fraction of the promotor region of the *IKBKG* gene (chrX:154546678-154547921) was calculated and visualized using Methylartist v1.2.7 [24].

## 3. Results

### 3.1. Clinical Description

The female individual was first brought in for evaluation at the age of 3 months. Pregnancy was unremarkable, and she was delivered spontaneously at a gestational age of 39 weeks and 2 days. Birth weight was 3728 g (0.83 z) [25], birth length was 53 cm (0.79 z) [25], and head circumference was 35 cm (0.24 z) [25]. Initially, she showed yellow purulent skin lesions at her left wrist and forearm as well as gluteal efflorescences. Due to breathing difficulties and cyanosis, she was admitted to neonatal intensive care unit the first night of her life and was discharged from the hospital without breathing difficulties 3 days later. After one day, she was readmitted due to poor drinking, hypothermia and circulatory centralization, with a suspected neonatal infection. Physical examination showed no signs of infection, but in the course, her skin showed yellow-white incrusted blisters, papules, and erythema on her upper and lower extremities. A consulted dermatologist discussed the possibility of IP. The skin lesions healed at approximately 3–4 months of age.

**Figure 2:**
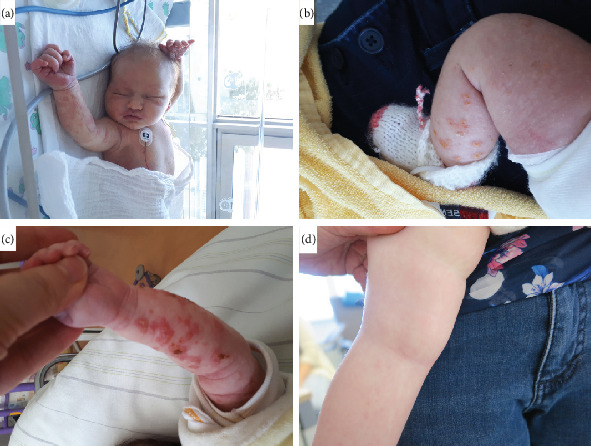
Individuals' skin manifestations. (a–c) Skin blisters on the upper and lower extremities and face. (d) Healed skin on the left leg at the age of 18 months, faint white maculae visible.

The child spoke her first words at the age of 15 months. She was able to sit with 12 months of age and was able to walk by the hand at the time of last examination in our genetic counselling (18 months of age). At that time, she was not able to walk freely or stand autonomously. She now weighed 9.85 kg (33 P) [26], her height was 78.8 cm (16 P) [26], her head circumference 46.5 cm (52 P) [26], and her BMI 15.9 kg/m^2^ (53 P) [26]. At her physical examination stages, one and two of her skin manifestations had already regressed and she showed very faint white maculae along the Blaschko lines in the groin and knee pit. She had no other systemic involvements at that time and was developing normally. Both the individual's mother as well as maternal grandmother also reported to have had skin lesions shortly after birth, but had no other symptoms throughout their lives. Only the index and the mother were available for testing (see Figure S1 and Figure 2).

### 3.2. Molecular Analysis

We performed WES to analyze comprehensively both SNVs as well as larger CNVs and simultaneously evaluate differential diagnoses. Due to the strong suspicion for IP, the *IKBKG* gene was our focus of interest, a parallel *IKBKG* MLPA remained inconspicuous (Figure S2A). Since variants in *IKBKG* are not called by the applied genomics software Varvis due to the confounding pseudogene, we manually inspected *IKBKG* using Integrative Genomics Viewer (IGV). We found a heterozygous likely pathogenic 5-bp deletion GRCh38/hg38: chrX:g.154556340_154556344del, NM_001099857.5(*IKBKG*):c.363_367del, p.(Leu122Glyfs⁣^∗^14) in Exon 3 of the *IKBKG* gene (Figure 1). Variant classification was based on the PVS1 and PM2 criteria (ACMG [16]).

**Figure 3:**
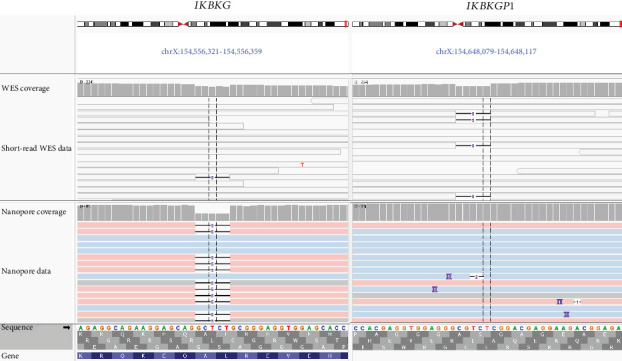
IGV screenshot of aligned short-read whole-exome sequencing reads and nanopore reads: The genomic region around the variant c.363_367del, p.(Leu122Glyfs⁣^∗^14) in the *IKBKG* gene (left) as well as the homologous region in the pseudogene *IKBKGP1* (right) in which none of the nanopore reads show the variant of interest. Nanopore reads are colored according to haplotypes. Transparent reads in WES data specify mapping quality of 0 indicating that reads cannot be mapped conclusively to a single region.

Segregation by Sanger sequencing using nonspecific primers (Table S1) confirmed the finding, albeit with skewed allelic ratio due to both *IKBKG* and *IKBKGP1* amplifications (Figure S2B). The variant was confirmed to be maternally inherited. Due to methodical limitations of short-read sequencing, we were not able to determine whether the variant was actually located on the *IKBKG* gene or its highly homologous pseudogene *IKBKGP1* and could therefore not conclusively confirm suspicion of IP (Figure S2B and Figure 3). Subsequently performed RNA analysis was meant to address this since *IKBKGP1* is not transcribed. Hence, only *IKBKG* transcripts would be amplified. However, neither of the two employed primer pairs for RNA analysis (Table S2) amplified sufficient quantities of the variant-carrying transcript for detection. Consequently, sequencing revealed only PCR products from the wild-type *IKBKG* transcript.

Next, we checked for skewed XCI in the blood of the proband and her mother. Pathogenic variants in *IKBKG* usually lead to skewed XCI due to negative selection against cells expressing the mutated allele [5]. XCI analysis investigating polymorphic repetitive elements in *AR* and *RP2* strengthened the suspicion of IP by showing extreme skewing in the mother of the proband with a ratio of 95:5 and a skewed XCI in the proband with a ratio of 85:15, consistent with the diagnosis. Subsequently conducted long-range PCR followed by sequencing of the proband's DNA confirmed the location of the variant on the *IKBKG* gene thereby affirming the diagnosis of IP in the proband's family.

We also incorporated ONT sequencing as part of the investigation process. To this end, we applied adaptive sampling targeting the q-arm of the X chromosome in the proband, thereby verifying the variant location in the *IKBKG* gene (Figure 3). Besides this, the ONT data was checked for further variants in the *IKBKG* gene in order to rule out genetic causes other than the above-described variant. All other SNVs identified via ONT (see Table S4) were excluded, either due to high frequency in the general population or due to unremarkable in silico predictions. These SNVs were not found in short-read WES data due to their location in intronic or regulatory regions. A CNV or SV affecting the *IKBKG* gene could also not be detected.

**Figure 4:**
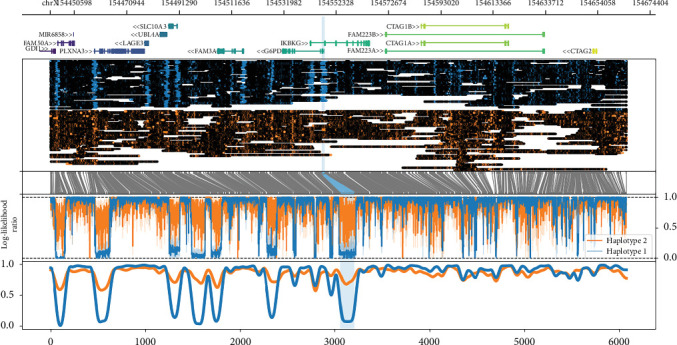
Haplotype-specific methylation profiles of the *IKBKG* locus showing increased methylation of the variant-carrying maternal allele (Haplotype 2, orange) compared to the wild-type paternal allele (Haplotype 1, blue). From top to bottom, this plot shows a gene track, haplotype-specific methylation calls (black color coded) relative to aligned read positions, a translation from genome space into a modified base space consisting only of instances of the methylated motif, the haplotype-specific methylation statistic (log-likelihood ratio—1: likely methylated, 0: likely unmethylated), and a smoothed sliding window plot showing the methylated fraction across the region (*IKBKG* gene ± 100 kb). The promoter region of the *IKBKG* gene is highlighted in light blue.

Furthermore, we used the ONT-generated 5mC methylation information to infer whether the variant in *IKBKG* is preferentially located on the activated or inactivated X chromosome through analysis of the haplotype-specific methylation of the two *IKBKG* promoters (see Figure 4). The methylated fraction in the promoter region of Haplotype 1 (paternal), which is designated as wild-type since it does not carry the variant in *IKBKG*, was 7.9%. In contrast, the methylated fraction of Haplotype 2 (maternal) carrying the variant in *IKBKG*, was 71.7%, suggesting a higher likelihood of being present on the inactivated X chromosome. These findings validate the previously mentioned skewed X-inactivation pattern in the individual and further confirm the mutated allele as the one preferentially undergoing inactivation. A methylation analysis of the *AR* and the *RP2* loci also confirmed the skewed XCI (Figure S3).

No relevant variants were identified through WES to account for the child's developmental delay, which is an uncommon finding in IP patients. A consultation with the attending physician confirmed that the child continues to receive neuropaediatric care. It cannot be ruled out that the developmental delay may be attributed to hypoxic brain injury resulting from cyanosis shortly after birth.

## 4. Discussion

The hallmarks of IP encompass the cutaneous manifestations generally characterized by four distinct stages that can overlap or may not develop at all in some patients [27], while other organs may be affected later in life. Here, we describe the case of an 18-month-old individual carrying a novel maternally inherited small deletion in *IKBKG*. Both the individual and the mother showed no signs of hyperpigmentation along the Blaschko lines and hypopigmented areas were very faint. The former is surprising, since hyperpigmented areas of the skin develop in as much as 98% of affected females and can persist up to the fourth decade of life [28], while hypopigmented skin areas have been suggested to be dramatically underreported in the past [27]. Furthermore, studies on genotype–phenotype correlations are very rare and suggest that variant type, affected domain, status of XCI, and genomic background may all contribute to the high phenotypic variability reported in IP patients ranging from mild skin alterations to severe central nervous system (CNS) abnormalities [7]. Interestingly, loss-of-function variants have been shown to coincide a higher likelihood of skewed or extremely skewed XCI [29] and skewing seems to negatively correlate with disease severity and CNS involvement [30]. Also, hypomorphic variants (variants that preserve some protein activity) show a greater involvement of different tissues compared to more severe protein function abolishing variants [29]. These findings support the effect of the small deletion we report here and offer an explanation to the rather mild, single-organ phenotype of the family. In confirmation, XCI status of the family showed extremely skewed XCI in the mother (95:5) and skewed XCI in the index patient (85:15), consistent with the notion that skewing is known to increase with age [31] and consistent with the above-mentioned correlations.

Diagnostic strategies for IP still heavily rely on long-range PCR and Sanger sequencing as the gold standard method for testing [15]. However, broad screening technologies such as WES are exceedingly employed in routine genetic laboratories. This short-read–based capture probe data analysis makes reliable discrimination between highly similar genes and pseudogenes difficult and identified variants need to be validated using an independent method. Recent innovations have tried to bypass this by optimizing the bioinformatics pipeline in order to mask the *IKBKGP1* pseudogene allowing only for detection of variants in *IKBKG* [32]. This approach could potentially serve as an alternative to the traditional long-read Sanger sequencing. In addition, since IP is an X-linked disorder, analysis of skewed XCI can serve as a diagnostic criterion, aiding in the determination of whether or not a variant might be causative for the patients' symptoms. Traditional analysis of fragment lengths after methylation sensitive restriction enzyme digest at the *AR* and *RP2* loci is frequently employed for analysis of skewed XCI. Most recently, this has also been achieved using ONT sequencing after CRISPR-Cas9 enrichment, demonstrating a series of advantages towards traditional diagnostic strategies and reliably and reproducibly determining the XCI status in multiple females [33]. Since ONT sequencing relies on long-reads and allows for pinpointing a variant's location within a gene, even in the presence of closely related pseudogenes, it can facilitate both variant mapping and XCI analysis, even extending to the precise assignment of the variant to the predominantly active or inactive X chromosome. This combination of features might render ONT sequencing a potent tool, particularly valuable in cases of X-linked disorders characterized by recognizable XCI skewing.

Here, WES revealed the presence of a small deletion in either *IKBKG* or *IKBKGP1*. Since mapping was inconclusive, and IP was specifically suspected by the physician, we visually explored the *IKBKG* locus using the IGV. Still, we were unable to assign the variant to either *IKBKG* or *IKBKGP1*. RNA analysis was meant to address this issue, since *IKBKGP1* is not transcribed; however, we were not able to amplify the variant-carrying transcript and sequenced wild-type transcripts only. At the time, we were not certain if this was due to the variant being located on the *IKBKGP1* locus or due to nonsense-mediated mRNA decay (NMD) (a physiological surveillance pathway degrading mutant mRNAs harboring PTCs [34, 35]) of *IKBKG* transcripts carrying the variant. Due to the inflicted frameshift and ultimate PTC of the detected variant as well as its location within the transcript, rapid degradation by NMD is highly likely, hence preventing it from detection via mRNA analysis through Sanger sequencing. Strategies to circumvent this diagnostic gap have been reported previously, mainly relying on pharmacological inhibition of NMD [36] that can be adapted to the requirements of diagnostic laboratories [37]. Given that XCI is skewed in the proband at an 85:15 ratio, transcription of the transcript harboring the variant is anticipated to occur in only 15% of cells, resulting in an allelic fraction (AF) of approximately 7.5%. Taking into account the effect of NMD, detection of the variant via Sanger sequencing becomes highly improbable, considering its established detection threshold of approximately 20% AF [38]. Therefore, we were only able to confirm the diagnosis of IP and the location of the variant on *IKBKG* by applying long-read PCR and Sanger sequencing. It needs to be mentioned that the long-range PCR was conducted in an external laboratory, for which we have not been able to obtain specific details regarding the methodical approach (e.g., primer sequence and binding location). Therefore, and in order to investigate ONT's potential as a possible diagnostic strategy in IP, we furthermore employed ONT sequencing and haplotype mapping of methylation profiles. To our knowledge, this is the first time ONT sequencing has been employed this way, opening up the possibility for its implementation as a second tier (after WES) or even a first tier diagnostic strategy when suspecting IP (and potentially further X chromosomal dominant disorders associated with skewing of XCI). ONT sequencing can provide two separate strands of information that support the diagnosis without the need for additional analyses, such as traditional XCI skewing analysis or RNA analyses, thereby saving both time and money in the process. Further analyses reiterating our findings are necessary.

This study underlines the importance of thorough investigation, exceeding the mere variant detection tools of standard genomics software, especially in cases with specific phenotypes and underlying genes with highly homologous pseudogenes. It also demonstrates, for the first time, the potential of ONT sequencing as a tool for precise variant mapping in the context of differentially methylated promoter regions due to skewed XCI.

## Data Availability

The data that support the findings of this study are available on request from the corresponding author. The data are not publicly available due to privacy or ethical restrictions. The genetic variant as well as its classification is available in the ClinVar repository, Variation ID: 2579743.

## Supporting Information

Supporting Information Additional supporting information can be found online in the Supporting Information section. Table S1: Primer pairs used for bidirectional Sanger sequencing. Table S2: RNA primer used for bidirectional Sanger sequencing. Table S3: Quality metrics of ONT sequencing. Table S4: Variants called in *IKBKG* based on ONT sequencing data. Figure S1: Pedigree. Figure S2: MLPA and Sanger results. Figure S3: Haplotype-specific methylation profiles of the *RP2* and the *AR* locus.
