# Supplementary material for: Incorporating Nanopore Sequencing Into a Diverse Diagnostic Toolkit for Incontinentia Pigmenti
Source: Hum Mutat. 2025 Jan 30;2025:6657400. doi: 10.1155/humu/6657400 (PMC12267976; doi:10.1155/humu/6657400)
Supplement: Supporting Information — Additional supporting information can be found online in the Supporting Information section. Table S1: Primer pairs used for bidirectional Sanger sequencing. Table S2: RNA primer used for bidirectional Sanger sequencing. Table S3: Quality metrics of ONT sequencing. Table S4: Variants called in IKBKG based on ONT sequencing data. Figure S1: Pedigree. Figure S2: MLPA and Sanger results. Figure S3: Haplotype-specific methylation profiles of the RP2 and the AR locus. [file 6657400.f1.docx]

**Supplementary material**

**Table S1:** Primer-pairs used for bidirectional Sanger-Sequencing

| **Primer** | **Sequence** | **Genomic position (GRCh38) on ChrX** | **Length of PCR product in bp** |
| --- | --- | --- | --- |
| Exon 3.1 Forward Primer | 5’-GAGCTTCTGCATTTCCAAGC-3’ | 154,556,209 | 324 |
| Exon 3.1 Reverse Primer | 5’-TAAACCGGAACTGGGAGTGT-3’ | 145,556,532 |  |
| Exon 3.2 Forward Primer | 5’-CTGGCCTCTGACTTCCTGAG-3’ | 154,556,066 | 392 |
| Exon 3.2 Reverse Primer | 5’-TTGGGGGACCCCGACCACGG-3’ | 154,556,457 |  |

**Table S2:** RNA-Primer used for bidirectional Sanger-Sequencing

| **Primer** | **Sequence** | **cDNA position on NM_001099857.5** | **Exon** | **Length of PCR product in bp** |
| --- | --- | --- | --- | --- |
| RNA Primer B F | 5’-CGGCAGCAGATCAGGACGTA-3’ | c.59 | 2 | 347 |
| RNA Primer B R | 5’-CATCTGCTGCTGGCATCTCT-3’ | c.405 | 3/4 |  |
| RNA Primer C F | 5’-G​G​C​A​C​C​T​C​T​G​G​A​A​G​A​G​C​C​A​A​C​-3’ | c.8 | 2 | 390 |
| RNA Primer C R | 5’-G​C​T​G​G​C​A​T​C​T​C​T​T​C​A​G​G​T​G​C​T​-3’ | c.397 | 3 |  |

**Table S3:** Quality metrics of ONT sequencing. The q-arm of the X chromosome was targeted by adaptive sampling

|  | **Total output** | **On-target output** |
| --- | --- | --- |
| **Total bases (Gb)** | 38.7 | 7.9 |
| **Coverage** | 13x | 86x |
| **Number of reads** | 30,320,788 | 1,004,647 |
| **Median read quality** | 16.8 | 18.9 |
| **Read length N50 (kb)** | 1.0 | 19.1 |

**Table S4:** Variants called in *IKBKG* based on ONT sequencing data. Due to the pseudogene, no variants were called in the short-read WES data. Relevant variant classified as likely pathogenic, highlighted in bold. Regarding the overall concordance between WES and ONT data, we compared variants called on the q arm of the X chromosome within regions sufficiently covered by WES (≥50x). A total of 743 variants were identified by both short-read and long-read sequencing, while 14 variants were exclusively detected by short-read WES and 50 variants only by long-read Nanopore sequencing. The observed discordance primarily resulted from increased homopolymer-associated errors in Nanopore data and the reduced mappability of short reads, including regions such as the *IKBKG* gene.

| **Position (hg38)** | **Ref** | **Alt** | **Zygosity** | **Type of variant** | **gnomAD v4.1.0 (allele count: het/homo/hemi)** | **CADD** |
| --- | --- | --- | --- | --- | --- | --- |
| chrX:154536951 | G | A | heterozygous | upstream gene | 4324/192/1510 | 0.1 |
| chrX:154538857 | G | C | homozygous | upstream gene | 0/0/0 | 0.4 |
| chrX:154543081 | T | C | homozygous | upstream gene | 71,940/20,026/21,919 | 0.2 |
| chrX:154543136 | A | C | homozygous | upstream gene | 110,742/0/2 | 0.2 |
| chrX:154553820 | A | G | homozygous | intronic | 0/0/0 | 2.2 |
| **chrX:154556337** | **GGCTCT** | **G** | **heterozygous** | **frameshift** | **0/0/0** | **NA** |
| chrX:154558768 | G | C | homozygous | intronic | 0/0/0 | 1.2 |
| chrX:154559514 | G | A | homozygous | intronic | 0/0/0 | 5.0 |
| chrX:154560134 | G | A | homozygous | intronic | 0/0/0 | 6.9 |
| chrX:154560328 | AC | A | heterozygous | intronic | 279,272/0/0 | NA |
| chrX:154563953 | C | T | homozygous | intronic splice region^1^ | 0/0/0 | 11.4 |
| chrX:154564829 | C | A | homozygous | 3’ UTR | 0/0/0 | 0.5 |
| chrX:154564863 | C | T | homozygous | 3’ UTR | 0/0/0 | 1.9 |
| chrX:154566050 | G | C | homozygous | downstream gene | 0/0/0 | 2.8 |

^1^ – spliceAI with delta scores of 0.11 or lower

**
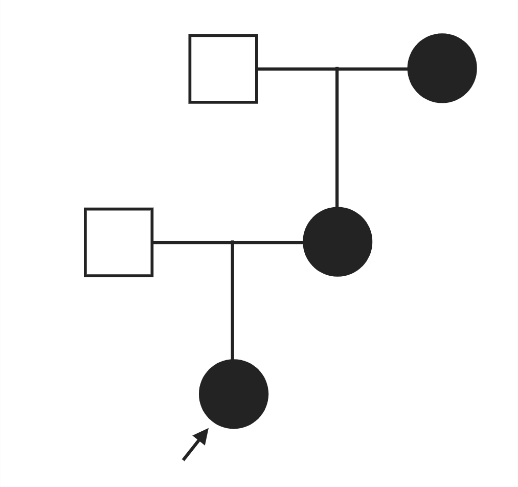
**

**Figure S1:** (Created with BioRender.com) Pedigree; affected female individual, mother and maternal grandmother with “skin lesions”; mother and index are both carriers of the *IKBKG* variant, the maternal grandmother was not tested

**
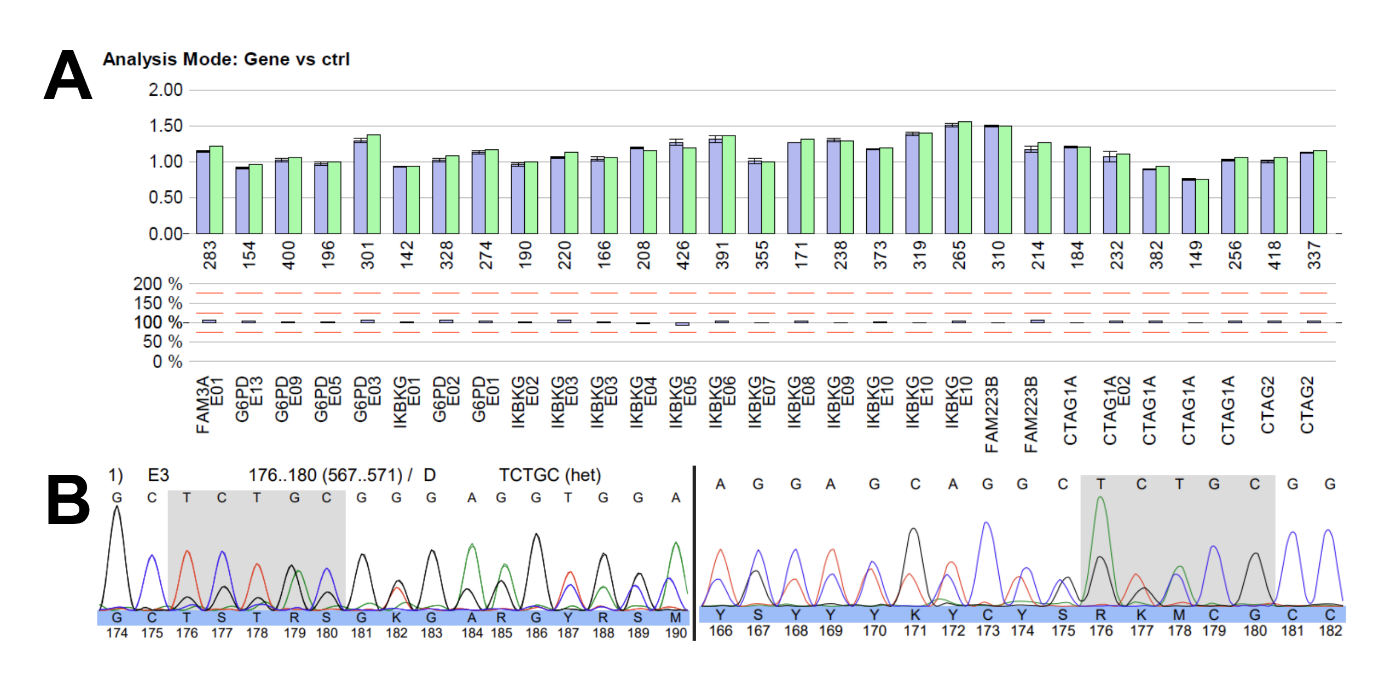
**

**Figure S2:** MLPA and Sanger results; **A** inconspicuous MLPA covering the whole *IKBKG* locus, however not the pseudogene; controls in blue, proband in green, all *IKBKG* exons 1-10 are covered **B** *left* forward primer (Exon 3.2 see Table S1) and *right* reverse primer, confirming a variant in either *IKBKG* or *IKBKGP1*, since primers are not *IKBKG* specific; results for primer Exon 3.1 (see Table S1) rendered similar results (not shown).

**Methylation status of the *RP2* and *AR* loci
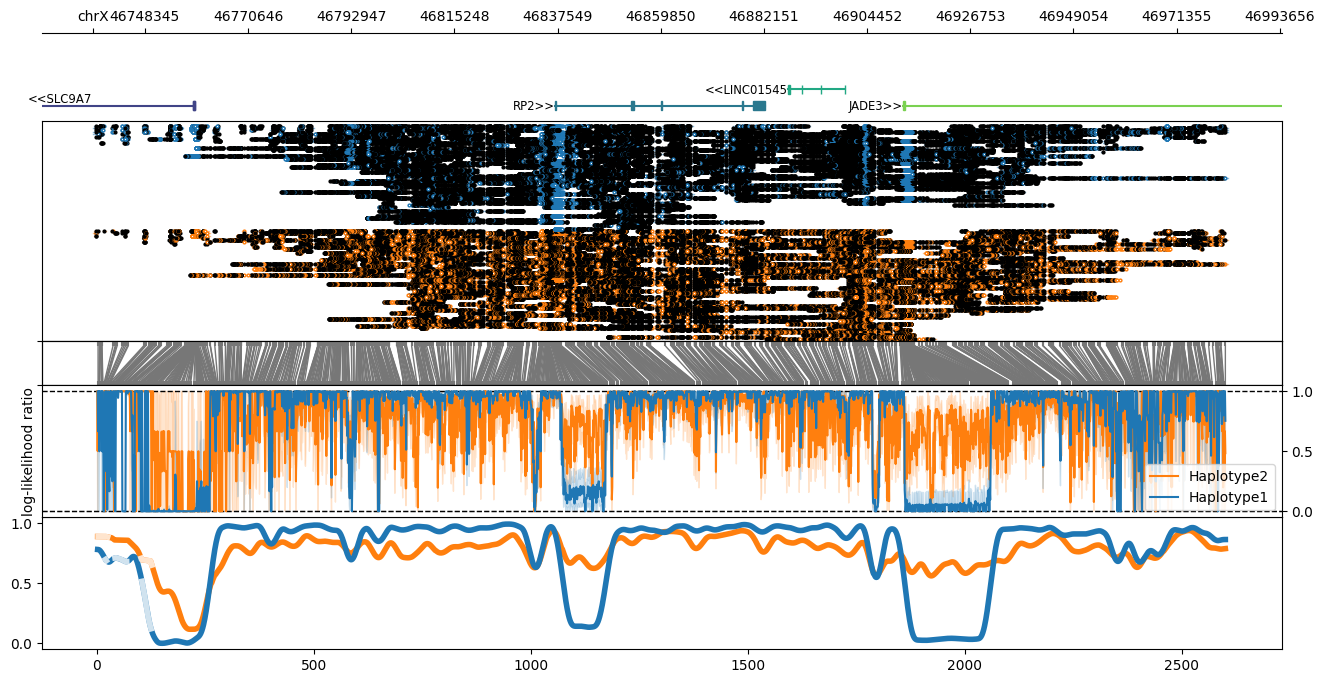
**

**
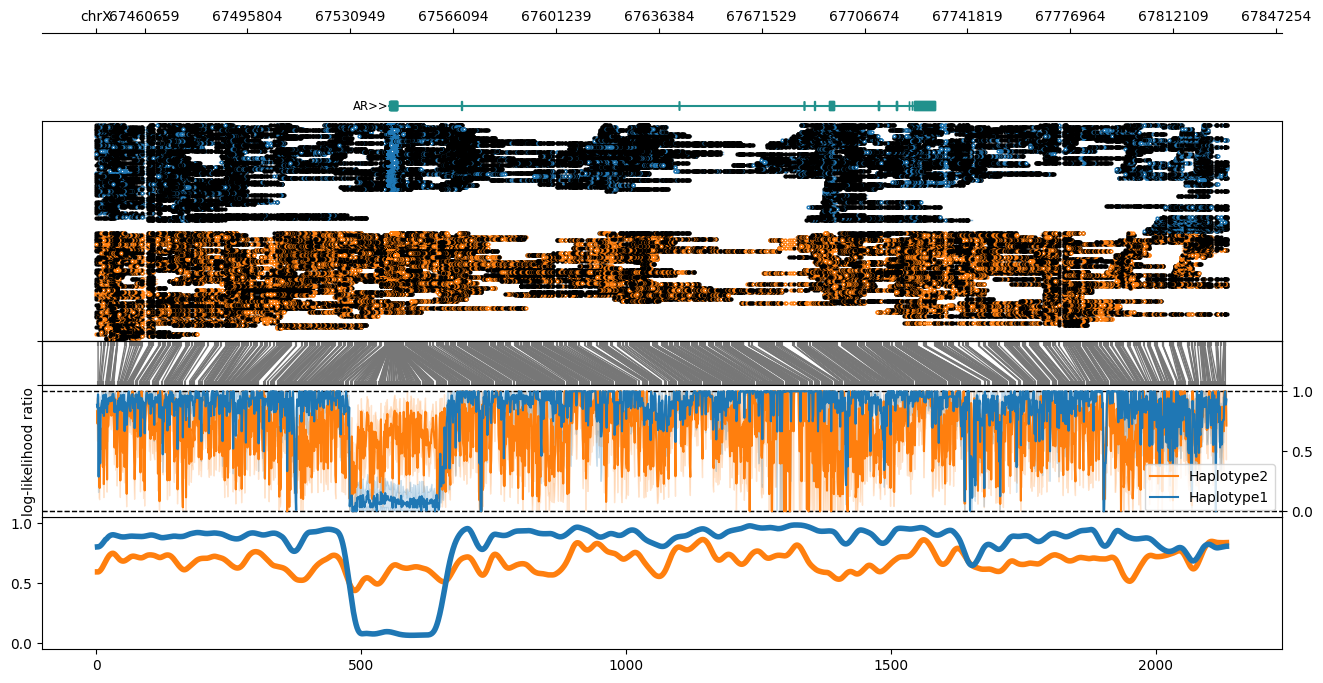
**

**Figure S3:** Haplotype-specific methylation profiles of the *RP2* (a) and the *AR* (b) locus showing differentially methylated haplotypes. In case of random XCI a similar methylation of both haplotypes would be expected. From top to bottom these plots show a gene track, haplotype-specific methylation calls relative to aligned read positions, a translation from genome space into a modified base space consisting only of instances of the methylated motif, the haplotype-specific methylation statistic (log-likelihood ratio) and a smoothed sliding-window plot showing methylation fraction across the region (*RP2/AR* gene ± 100 kb).
